# Fulminant inflammatory demyelination presenting as stroke‐in‐evolution in an elderly subject

**DOI:** 10.1002/brb3.1967

**Published:** 2020-12-08

**Authors:** Simone Sacco, Ilaria Callegari, Isabella Canavero, Elisa Coloberti, Lisa Maria Farina, Sabrina Ravaglia, Anna Simoncelli, Anna Pichiecchio, Giuseppe Micieli

**Affiliations:** ^1^ Department of Clinical Surgical Diagnostic and Pediatric Sciences Institute of Radiology University of Pavia Pavia Italy; ^2^ Neuroscience Consortium Monza Policlinico and Pavia Mondino University of Pavia Pavia Italy; ^3^ Emergency Neurology Unit IRCCS Mondino Foundation Pavia Italy; ^4^ Neuroradiology Unit IRCCS Mondino Foundation Pavia Italy; ^5^ Diagnostic Radiology Interventional Radiology and Neuroradiology Unit Fondazione IRCCS Policlinico San Matteo Pavia Italy; ^6^ Department of Brain and Behavioral Sciences University of Pavia Pavia Italy; ^7^ Present address: Department of Neurology UCSF Weill Institute for Neurosciences University of California San Francisco CA USA; ^8^ Present address: Department of Biomedicine University Hospital Basel University of Basel Basel Switzerland

**Keywords:** ADEM, fulminant inflammatory demyelination, inflammatory demyelinating disorders, tumefactive demyelinating lesions

## Abstract

**Background:**

Fulminant inflammatory demyelination is a possible presentation of inflammatory demyelinating disorders, thus representing a potential stroke mimic especially in younger patients.

**Aims of the study:**

To describe clinical and diagnostic pitfalls in a case of fulminant inflammatory demyelination presenting with stroke‐like symptoms in an elderly patient.

**Methods:**

Case report and case‐based review of the literature.

**Results:**

A 67‐year‐old woman, who accessed the emergency room as suspect stroke for hyperacute onset of rapidly worsening speech impairment and drowsiness, was later diagnosed with a huge brain inflammatory demyelination. Clinical, laboratory, and neuroimaging tests did not allow to put a more specific diagnosis. Due to the rapidly deteriorating course, she received immunosuppression with benefit.

**Conclusion:**

This report is meant to highlight the diagnostic challenges connected with fulminant inflammatory demyelination, which sometime can resemble a stroke‐in evolution and appear clinically unfitting for inclusion in any specific pathological entities within the broad‐spectrum of inflammatory demyelinating disorders.

## Graphical abstract

Fulminant inflammatory demyelination represents a possible presentation of inflammatory demyelinating disorders. Here we describe a case affecting a 67 year‐old woman, who accessed the emergency room for hyperacute onset of rapidly worsening speech impairment and drowsiness. This report is meant to highlight the diagnostic challenges connected with fulminant inflammatory demyelination, which sometime can resemble a stroke‐in evolution and appear clinically unfitted for inclusion in any specific pathological entities within the broad spectrum of inflammatory demyelinating disorders.

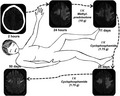

## 1 BACKGROUND

Inflammatory demyelinating disorders (IDD) represent a potential, although uncommon, stroke mimic, which should always be considered in the emergency department (ED) setting, particularly in young subjects showing acute onset of focal neurological deficits (Gibson & Whiteley, 2013).

Acute‐onset IDD can indeed be associated with rapid neurological deterioration configuring a clinical entity defined as fulminant inflammatory demyelination (FID). FID, which can be expression of several pathological entities within central nervous system (CNS) IDD spectrum, is classically associated with poor outcome, but prompt recognition through MRI and early, aggressive treatment can improve the prognosis (Bevan & Cree, 2015).

Here, we describe a case of FID in an elderly woman, where the hyperacute onset and the rapid worsening of symptoms resembled a stroke‐in evolution.

## 2 CASE REPORT

**FIGURE 1:**
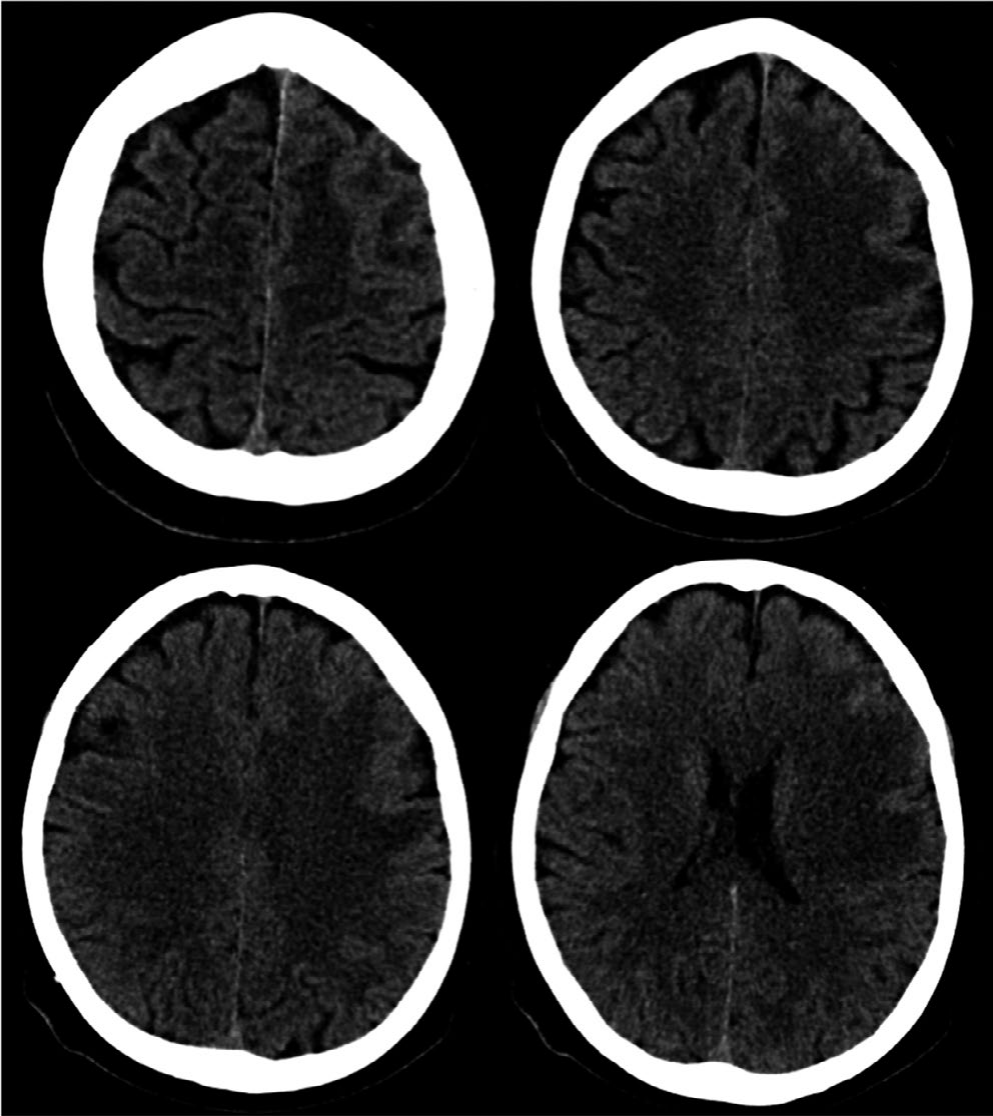
Brain CT performed at the ED, revealing in the left hemisphere an extensive, pale hypodensity with finger‐like borders associated to flattened cerebral sulci*

A 67‐year‐old woman, without remarkable medical and neurological history, accessed the ED for acute onset of speech impairment and drowsiness. Clinical assessment revealed moderate hypertension (165/85 mmHg) and mild leukocytosis (12.1 × 10^3^/microL; r.v. 4.8–10.8) without fever. Neurological examination, at 2 hr from onset, revealed normal alertness, nonfluent aphasia and moderate motor and sensory right‐side impairment (NIHSS 9). In the suspect of a left middle cerebral artery stroke, a brain CT was performed, detecting a wide, pale hypodensity, with finger‐like borders and flattened cerebral sulci in the left hemisphere (Figure 1). Intra and extra‐cranial vessels CT angiography resulted normal excluding eligibility for reperfusion treatments and leading to hospitalization. Within 24 hr from the onset, the patient showed rapid neurological deterioration, up to comatose state and right hemiplegia‐hemianesthesia (GCS = 5, NIHSS = 25).*

**FIGURE 2:**
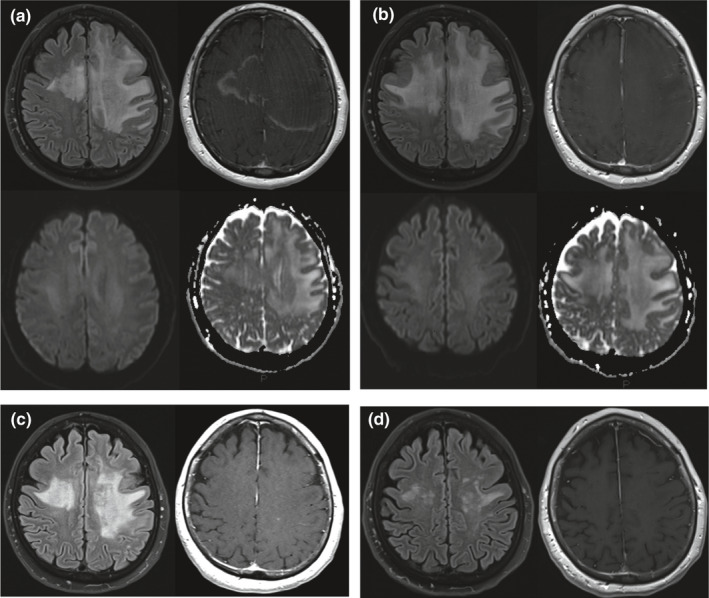
Axial FLAIR and postcontrast T1w images at different time points: additionally, for A and B, DWI trace and ADC sequences are shown. (a) 24 hr after symptoms onset: diffuse hyperintense alteration involving the left frontal lobe and deep white matter of the right frontal lobe, associated with a peripheral ring of contrast enhancement; there is no evidence of restricted diffusion on DWI trace and ADC sequences. (b) 11 days after symptoms onset (after conclusion of intravenous methylprednisolone treatment): disappearing of peripheral ring of contrast enhancement with progression of hyperintense alteration now partially involving the left parietal lobe and, more extensively, the right frontal lobe; there is no evidence of restricted diffusion on DWI trace and ADC sequences. (c) 25 days after symptoms onset (after first cyclophosphamide administration): initial reduction in size of T2 hyperintense alteration, without contrast enhancement. (d) 50 days after symptoms onset (after second cyclophosphamide administration): further reduction in size of T2 hyperintense alteration without contrast enhancement*

Anamnestic revision detected symptoms of upper respiratory tract infection with slight fever and headache, during the preceding 3 days. Cerebrospinal fluid (CSF) examination showed mild pleocytosis (11 lymphocytes/mm^3^, r.v. <2), hyperproteinorrachia (albumin 63 mg/dl, r.v. 10–30), and “mirror pattern” oligoclonal bands. An extensive infective screening on blood and CSF (bacterial and mycobacterial cultures, PCRs/antibody testing for HIV, HSV1‐2, JCV, Enterovirus, VZV, West Nile virus, Panflavivirus, Mycoplasma, Legionella, and Pneumococcus) resulted negative. A broad‐spectrum, empirical therapy with ceftriaxone and acyclovir was started. No CNS‐specific autoantibodies (anti‐AQP4, anti‐MOG; onconeural panel: anti‐ Hu, Ma/Ta, GAD, amphiphysin, CV2/CRMP5; autoimmune encephalitis panel: anti‐NMDA‐R, LG1, CASPR2, GABA‐A and B, AMPA, Glu‐R1, Gly‐R1, and D2) were detected. Blood and CSF cellular immunophenotypes were normal without signs suggesting either systemic or CNS‐specific hematological disorders. Brain MRI, performed 24 hr after symptoms onset, showed, within the anterior portion of the left hemisphere white matter (WM), a diffuse T2‐hyperintense alteration, which involved the corpus callosum and spared the cortical gray matter. No restricted diffusion was detectable within the lesion. After gadolinium administration, a peripheral open‐ring pattern of contrast enhancement suggestive for IDD was detected (Figure 2a). No other brain alterations suggestive for demyelinating lesions were detected. No spinal cord involvement was detected by spine MRI and somatosensory evoked potentials. EEG excluded epileptic activity.

Treatment with methylprednisolone 1 g daily was started within 48 hr from onset and continued for 10 days, without any clinical improvement. Follow‐up MRI showed a widening of T2‐hyperintense alterations (Figure 2b), suggesting the need for a second‐line treatment. A single dose of cyclophosphamide (1 g/m^2^ body surface = 1.75 g, i.v.) was thus administrated. After 10 days, the patient showed a notable neurological improvement, with full recovery of consciousness, soon followed by partial improvement of verbal fluency and sensory‐motor deficits. After 35 days from the first, a second dose of cyclophosphamide (1.75 g, i.v.) was administered. A substantially complete clinical recovery was rapidly reached, and no other treatment was applied. Follow‐up neurological examinations after 1 month, 1 and 2 years revealed only residual right‐side pyramidal signs. Follow‐up MRIs revealed progressive improvement with almost complete resolution of previously described findings (Figure 2c,d). Over a 2‐year observation, no new brain or spinal cord alterations were detected and the subject did not experience neurological relapses or clinical worsening. Due to the favorable, monophasic course and the disappearance of the cerebral lesions at follow‐up MRIs, cerebral biopsy was not performed.*

## 3 DISCUSSION

The goal of the present report is to emphasize the diagnostic challenge connected with FID, particularly when affecting elderly. Whereas the hyperacute onset of focal neurological signs and the age of our patient initially suggested a cerebrovascular disorder, the subsequent clinical course and the neuroradiological features of the lesion oriented toward a possible IDD.

The prototypical IDD is represented by multiple sclerosis (MS), which generally affects young subjects showing space‐ and time‐disseminated WM lesions (Thompson et al., 2018). Uncommonly, certain subjects might show, since from the onset, atypical WM lesions with size >2.5 cm and mass effect (Wallner‐blazek et al., 2013) sometimes associated with a monophasic course of disease. The terms referring to these conditions are heterogeneous and range from rare MS variants such as tumefactive MS (TMS), Marburg's MS (MVMS), Balo's concentric sclerosis (BCS) to other distinct pathological entities with a presumable autoimmune inflammatory origin, such as acute disseminated encephalomyelitis (ADEM) (Bevan & Cree, 2015). Other Authors (Poser et al., 1992; Rahmlow & Kantarci, 2013) introduced the encompassing concept of tumefactive demyelinating lesions (TDL), defined as solitary large signal abnormalities associated with mass effect, perilesional edema, and/or ring enhancement, which may represent atypical presentation of the abovementioned IDD and a stand‐alone diagnosis. Clinical presentation includes headache, encephalopathy signs, and focal cortical signs depending on lesion size and location.

Besides the stroke‐mimic deception we faced in the ED, the diagnostic challenge of the present case is represented by the difficulty in formulating a definite diagnosis based on clinic‐ radiological data in the absence of histo‐pathological assessment. The clinical course during the 2‐year follow‐up period, coherently with the negative hematological tests performed at onset, helped to exclude cerebral lymphoma from the neuroradiological differential diagnosis (Chiavazza et al., 2018). Furthermore, no diffusion restriction was indeed evident at any time‐point within the lesion described, making less likely the possibility of lymphoma and ruling out the possibility of a stroke outside of the ED setting. It is, however, important to remember that in FID there can be association of gadolinium enhancement and restricted diffusion, with the former sometime chronologically following the latter (Hyland et al., 2013) BCS was excluded by the lack of the classical “onion‐like” MRI appearance, showing alternated iso‐ and hyperintense rings on T2‐weighted imaging, likely as expression of layers with relatively preserved or lost myelin (Bevan & Cree, 2015). MVMS could as well be excluded given the absence of progressive deterioration overtime, usually leading to death within months from onset (Bevan and Cree, 2015). TMS mostly occurs during the 2nd and 3rd decade (Lucchinetti et al., 2008), thus is atypical in the elderly. In our case, the lack of previous neurological history and pre‐existing demyelinating lesions, as well as the 2‐year follow‐up, ruled out MS.*

Acute disseminated encephalomyelitis affects mainly children and young individuals, with an incidence of 0.6 per 100,000 per year. This diagnosis is rare among young adults and almost exceptional beyond the age of 65, with only few cases reported in literature (Wang et al., 1996; Kaunzner et al., 2017). No criteria, indeed, have ever been established for diagnosis among adults. ADEM usually follows infections or vaccinations (Pohl et al., 2016) and occurs within 6 days to 6 weeks from an antigenic challenge; overlapping of neurologic and infective symptoms, as here described, is unusual and may point to a parainfective, rather than postinfective process. It is well known, however, that up to 26% of ADEM lacks of clear prodromal manifestations (Tenembaum et al., 2002).

Typically, ADEM patients develop neurological deficits sub‐acutely, conveying to peak within 2–5 days instead of the 24 hr here reported. Aggressive variants, such as Hurst's disease, present neurological deterioration within hours (Rahmlow & Kantarci, 2013) but are associated with hemorrhagic lesions, which were never detected in our patient.

Our report highlights the difficulty in defining a clear‐cut diagnosis in FID presenting some clinical features of multiple pathological entities (TDL, TMS, ADEM, Hurst's disease, parainfective demyelination) but also some relevant atypia precluding a conclusive labeling. The outcome‐arranged agreement to overlook pathological examination, deriving from a thoughtful risk‐benefit evaluation, contributed to this diagnostic pitfall (Hardy et al., 2016).

First‐line treatment with high‐dose intravenous steroids may show scarce efficacy, especially in IDD with atypical presentation (Jaskowiak, 2016). In these cases, progressive neurological and radiological deterioration can justify aggressive immunosuppression strategies (Berzero et al., 2016; Rahmlow and Kantarci, 2013). Plasma exchange is generally considered the second‐line therapy for FID patients not responding to corticosteroids (Hardy and Chataway, 2013), especially children and in patients with ring‐enhancing lesions and mass effect (Magaña et al., 2011), and however, given the rarity of the disease, no randomized controlled clinical studies are available and there are no evidences for effect on eventual disease reactivation. Other therapeutic options include immunosuppressors such as cyclophosphamide (Berzero et al., 2016; Rahmlow & Kantarci, 2013) that in our patient was associated with an impressive recovery. It is unclear, however, whether the recovery was associated with the treatment rather than the disease's nature itself. Clinical recovery is indeed frequent in ADEM, and follow‐up imaging demonstrates, in the majority of cases, partial or complete resolution usually conveying to a monophasic course of disease (Rahmlow and Kantarci, 2013; Tenembaum et al., 2002).

Fulminant inflammatory demyelination is usually not included among stroke differential diagnosis (Gibson & Whiteley, 2013). Nonetheless, a recent case series described the stroke‐mimic, fulminant course of pathologically proven ADEM affecting 5 subjects with age ranging from 57 to 85 (Tenembaum et al., 2002). The authors suggested that an exceptionally fulminant course in this population may be due to an age‐related decrease in repair efficacy and oligodendrocyte response.

## 4 CONCLUSION

Among the broad IDD spectrum, several pathological entities can present as FID. Specific IDD such as ADEM might show a particular fulminant course among elderly subjects. When dealing with hyperacute onset and rapidly worsening focal neurologic signs, once vascular etiology has been excluded, it is therefore always advisable considering FID among differential diagnoses. The diagnostic work‐up remains difficult for the presence of many partially and potentially overlapping IDDs often hindering clinical management. In the presence of severe and progressive neurological deterioration, not responsive to first‐line treatments, early, and aggressive immunosuppressive therapy can be considered.

## CONFLICT OF INTEREST

None to declare.

## AUTHOR CONTRIBUTION

All authors participated in the study and manuscript editing and take public responsibilities for the manuscript contents. 1) Simone Sacco and Ilaria Callegari involved in analysis and interpretation of data, drafting the article and revising it critically. 2) Isabella Canavero involved in acquisition, analysis and interpretation of data, coordination of contributors, and critical revision of the article. 3) Elisa Coloberti, Sabrina Ravaglia, Anna Simoncelli, and Lisa Maria Farina: involved in acquisition of data and critical revision of the article. 4) Anna Pichiecchio and Giuseppe Micieli involved in conception and design, acquisition, analysis and interpretation of data, and critical revision of the article.

## ETHICAL APPROVAL

Written informed consent was obtained from the patient.

### PEER REVIEW

The peer review history for this article is available at https://publons.com/publon/10.1002/brb3.1967.

## Data Availability

Complete clinical data that support the findings of this study are available on request from the corresponding author. The data are not publicly available due to privacy restrictions. Other data sharing is not applicable to this article as no datasets were generated or analyzed during the current study (case report and case‐based narrative review).

## Footnotes

^*^
Corrections added on March 18, 2021, after first online publication: The text “the following” was removed from the first paragraph in Section 2; the text “ at the MRI” was removed from the last paragraph in Section 2; Figures 1 and 2 were updated; the citation “(Bevan and Cree, 2015)” was added near the end of the third paragraph in Section 3.
